# Second generation of pepino mosaic virus vectors: improved stability in tomato and a wide range of reporter genes

**DOI:** 10.1186/s13007-019-0446-4

**Published:** 2019-05-28

**Authors:** Fabiola Ruiz-Ramón, Raquel N. Sempere, Eduardo Méndez-López, M. Amelia Sánchez-Pina, Miguel A. Aranda

**Affiliations:** 1 Present Address: R + D+I Department, Abiopep S.L., Murcia, Spain; 2 Centro de Edafología y Biología Aplicada del Segura (CEBAS), Consejo Superior de Investigaciones Científicas (CSIC), Murcia, Spain

**Keywords:** BAR, DsRed, mCherry, Potexvirus, Viral factory, VRC, Viral replication complex

## Abstract

**Background:**

Vectors based on plant viruses are important tools for functional genomics, cellular biology, plant genome engineering and molecular farming. We previously reported on the construction of PepGFP2a, a viral vector based on pepino mosaic virus (PepMV) which expressed GFP efficiently and stably in plants of its experimental host *Nicotiana benthamiana*, but not in its natural host tomato. We have prepared a new set of PepMV-based vectors with improved stability that are able to express a wide range of reporter genes, useful for both *N. benthamiana* and tomato.

**Results:**

We first tested PepGFPm1 and PepGFPm2, two variants of PepGFP2a in which we progressively reduced a duplication of nucleotides encoding the N-terminal region of the coat protein. The new vectors had improved GFP expression levels and stability in *N. benthamiana* but not in tomato plants. Next, we replaced *GFP* by *DsRed* or *mCherry* in the new vectors PepDsRed and PepmCherry, respectively; while PepmCherry behaved similarly to PepGFPm2, PepDsRed expressed the reporter gene efficiently also in tomato plants. We then used PepGFPm2 and PepDsRed to study the PepMV localization in both *N. benthamiana* and tomato cells. Using confocal laser scanning microscopy (CLSM), we observed characteristic fluorescent bodies in PepMV-infected cells; these bodies had a cytoplasmic localization and appeared in close proximity to the cell nucleus. Already at 3 days post-agroinoculation there were fluorescent bodies in almost every cell of agroinoculated tissues of both hosts, and always one body per cell. When markers for the endoplasmic reticulum or the Golgi apparatus were co-expressed with PepGFPm2 or PepDsRed, a reorganisation of these organelles was observed, with images suggesting that both are intimately related but not the main constituents of the PepMV bodies. Altogether, this set of data suggested that the PepMV bodies are similar to the potato virus X (PVX) “X-bodies”, which have been described as the PVX viral replication complexes (VRCs). To complete the set of PepMV-based vectors, we constructed a vector expressing the *BAR* herbicide resistance gene, useful for massive susceptibility screenings.

**Conclusions:**

We have significantly expanded the PepMV tool box by producing a set of new vectors with improved stability and efficiency in both *N. benthamiana* and tomato plants. By using two of these vectors, we have described characteristic cellular bodies induced by PepMV infection; these bodies are likely the PepMV VRCs.

## Background

Vectors based on plant viruses are important tools for functional genomics, cellular biology, plant genome engineering and molecular farming [1–6]. Different strategies have been used to achieve stable and efficient recombinant gene expression from modified plant virus genomes, including gene substitution, gene insertion, modular or deconstructed systems and peptide display fusion [4, 7–9]. These approaches have been applied to some of the most popular plant viruses modified to function as expression vectors, including tobamoviruses, potexviruses, tobraviruses, geminiviruses and comoviruses [10].

We have previously reported on the construction of a set of viral vectors based on pepino mosaic virus (PepMV) [11]. PepMV (species *Pepino mosaic virus*, genus *Potexvirus*, family *Alphaflexiviridae*) has a small single-stranded positive-sense RNA genome of approximately 6.4 Kb which encodes five open reading frames (ORFs) flanked by 5′ and 3′ untranslated regions (UTRs) [12]. ORF 1 encodes a 164 KDa replicase protein (RdRp) that includes three functional domains, a methyl transferase, a helicase and an RNA polymerase domain [13–15], followed by three overlapping ORFs (2–4) which encode the triple gene block (TGB) proteins 1–3 (TGB1 of 26 kDa, TGB2 of 14 kDa and TGB3 of 9 kDa); TGB proteins are involved in cell-to-cell movement, suppression of RNA silencing and architecture of the viral factories [16–19]. ORF 5 encodes the 25 kDa coat protein (CP) required for encapsidation, cell-to-cell movement and suppression of RNA silencing as well [13, 19–21]. In our previous work, up to three different strategies were used to develop the PepMV-based vectors, including substitution of the *CP* gene and duplication of the *CP* subgenomic mRNA promoter; in the first case, the vector was unable to move out of the agroinoculated cells, in the second case the insert had poor stability, as it was easily lost during plant to plant passages. The most stable PepMV vector was PepGFP2a, in which the transgene was expressed as a *CP* fusion through the self-cleaving 2A peptide from foot-and-mouth disease virus (FMDV). PepGFP2a was stable in plant to plant passages in *N. benthamiana* [11], but not in tomato, which is a principal host for PepMV. In the present report, we show results on the construction of a second generation of PepMV-based vectors, for which we wanted to improve their stability and usefulness both in the experimental host *N. benthamiana* and in tomato. We have also used our vectors to gain insights into the distribution of PepMV in *N. benthamiana* and tomato cells. For potato virus X (PVX), the type member of the genus *Potexvirus*, infection results in the formation of cytoplasmic “X-bodies” in the periphery of the cell nucleus. X-bodies are inclusion structures induced by the virus that function as viral replication complexes (VRCs). PVX viral proteins TGB1 to 3 play the main roles in VRC architecture, with TGB1 remodelling the actin and host endomembrane system, thereby contributing to the compartmentalization of infection [17] and TGB2/3 becoming inserted into membranous complexes and recruiting TGB1 and CP to the VRC [16, 22]. In contrast to PVX, the potexvirus alternanthera mosaic virus (AltMV) organizes its VRCs in chloroplasts [23]. For PepMV, Minicka et al. [24] carried out an ultrastructural analysis of tomato tissues infected with different isolates; no clear evidence was produced on the possible nature and localization of PepMV VRCs, even though different cytopathological alterations were observed. Using our PepMV vectors, we have observed cytoplasmic aggregates induced by the virus, normally in the vicinity of the cell nucleus, which may constitute the PepMV VRCs.

## Results

### Improvement of PepMV-based vectors for stable GFP expression

**Fig. 1:**
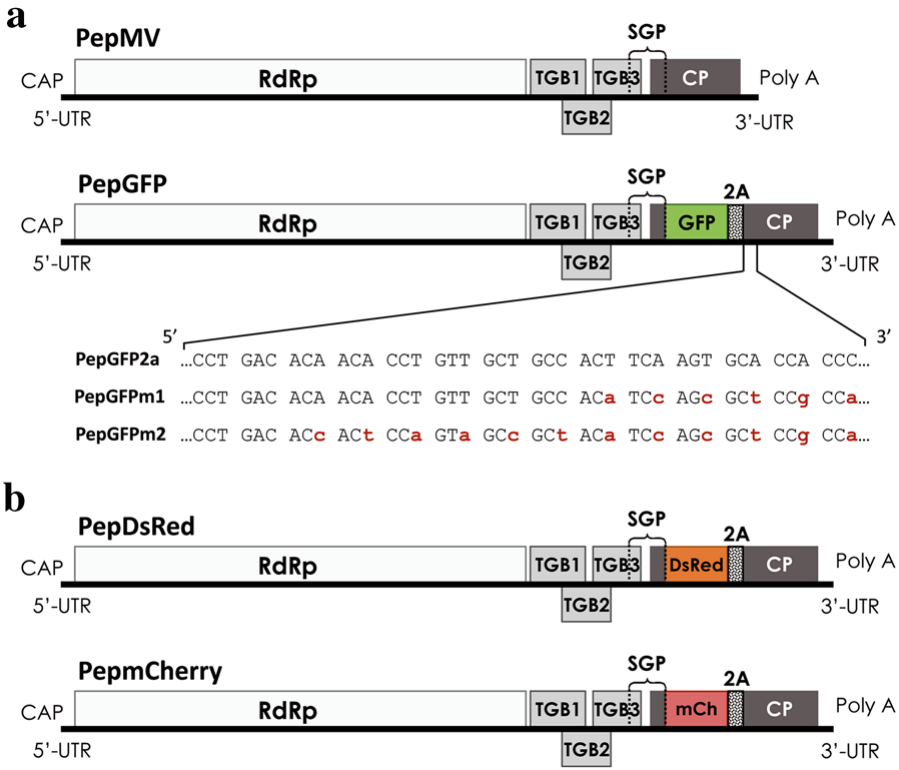
New PepMV vectors expressing the fluorescent proteins (FPs) GFP, DsRed and mCherry. The FPs were expressed as a CP fusion through the foot and mouth disease virus 2A catalytic peptide sequence. **a** Schematic representation (not to scale) of the PepMV genome and modified variants PepGFP2a, PepGFPm1, PepGFPm2, carrying the *GFP* gene. The nucleotides marked in red correspond to synonymous mutations introduced in the corresponding vector versions to avoid sequence duplications. **b** Schematic representation (not to scale) of the PepMV genome variants PepDsRed and PepmCherry carrying the *DsRed* and *mCherry* genes. RdRp: RNA dependent RNA polymerase; TGB1, TGB2 and TGB3: Triple gene block proteins 1–3; CP: Coat protein; SGP: CP subgenomic promoter

Our previous work showed that the insertion of a *GFP* transgene in vector PepGFP2a remained stable when infecting *N. benthamiana,* but not tomato plants [11; our unpublished results]. In this vector, the *GFP* coding sequence was fused in frame to the *CP* gene through the foot and mouth disease virus (FMDV) sequence encoding the 2A catalytic peptide [11]. Given that the promoter of the CP subgenomic RNA extends downstream of the AUG start codon, the introduction of a sequence duplication in PepGFP2a was necessary in order to keep the *CP* coding sequence intact [11] (Fig. 1a). To avoid losing the transgene during infection by homologous recombination between duplicated sequences, two new constructs were designed using pBPepGFP2a as the backbone, named pBPepGFPm1 and pBPepGFPm2. For pBPepGFPm1, six synonymous mutations were introduced, one in each of the last six nucleotide triplets of the duplicated sequence, while for pBPepGFPm2 the last twelve nucleotide triplets were mutated to further reduce sequence duplication (Fig. 1a).

**Fig. 2:**
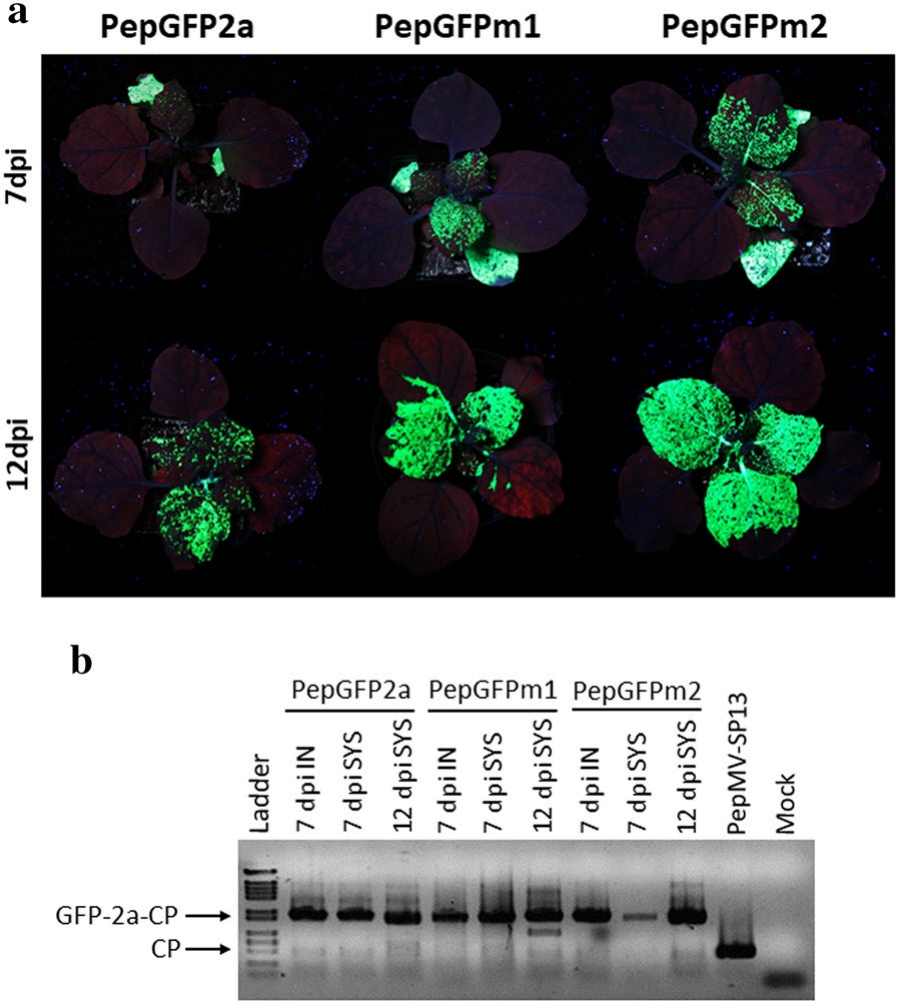
*N*. *benthamiana* plants infected with PepMV vectors expressing GFP. **a** Fluorescence in *N. benthamiana* plants inoculated with PepGFP2a, PepGFPm1 or PepGFPm2 under UV light at 7 and 12 d post inoculation (dpi). **b** Agarose gel electrophoresis of RT-PCR products from individual plants to check insert stability in PepMV vectors at 7 and 12 dpi. A minimum of 6 plants were used per treatment, with each treatment replicated a minimum of 8 times. All infected plants showed systemic fluorescence at 7 dpi. IN: Inoculated leaves; SYS: Systemically infected leaves; PepMV-Sp13, wild type virus

**Fig. 3:**
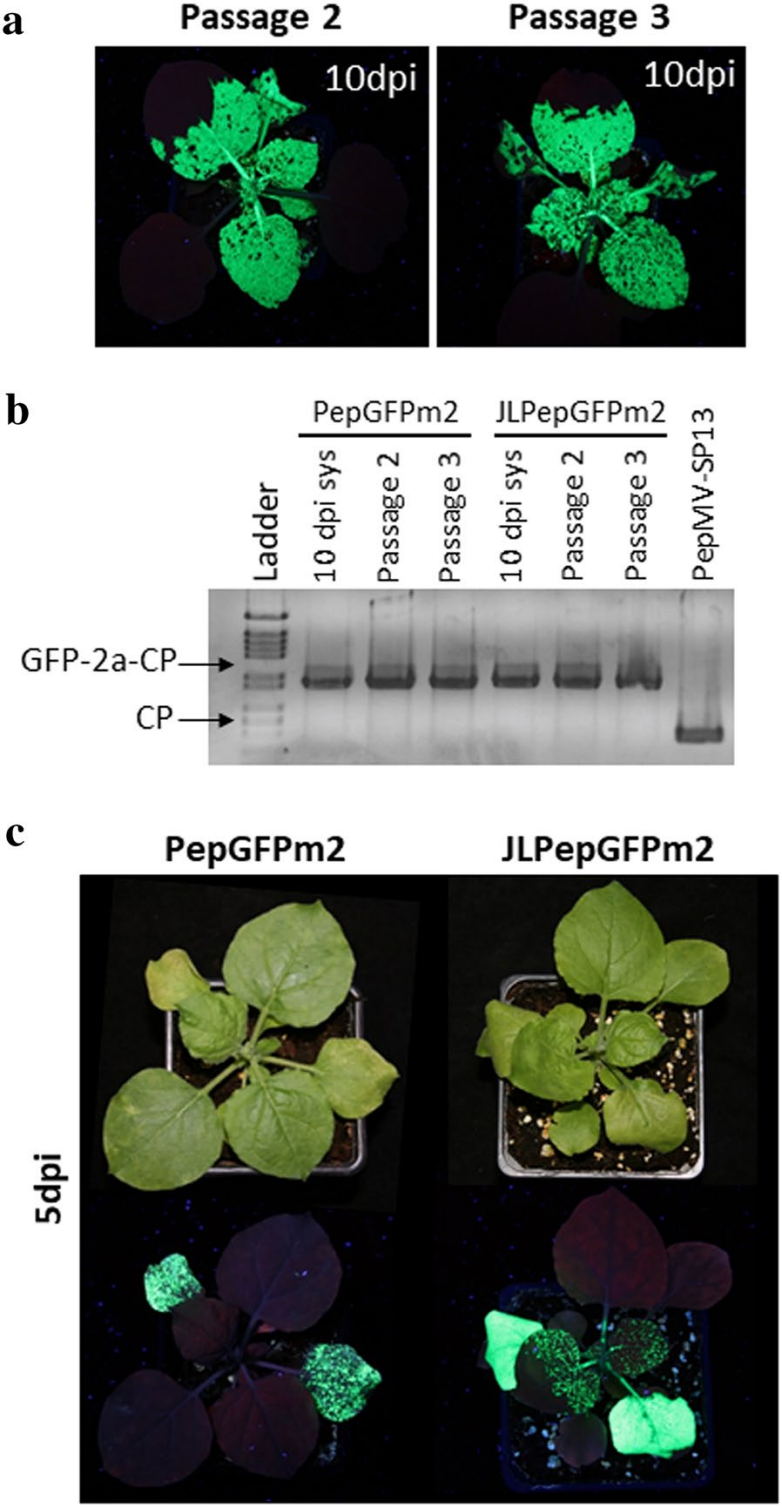
PepGFPm2 and JLPepGFPm2 vectors and their stability in plant-to-plant passages. **a** GFP expression from PepGFPm2 at 10 dpi after the second and third passage in *N. benthamiana* plants. **b** Agarose gel electrophoresis of RT-PCR products from individual plants to check insert stability during PepGFPm2 passages in *N. benthamiana* plants; samples were taken at 10 d post inoculation (dpi). **c** GFP expression in *N. benthamiana* plants inoculated with PepGFPm2 and JLPepGFPm2 vectors at 5 dpi. A minimum of 6 plants were used per treatment, with each treatment replicated a minimum of 5 times. All infected plants showed systemic fluorescence at 7 dpi. SYS: Systemically infected leaves; PepMV-Sp13, wild type virus

Stability assays were first performed on *N. benthamiana* plants. Leaves were agroinfiltrated with *A. tumefaciens* transformed with pBPepGFP2a, pBPepGFPm1 or pBPepGFPm2 in the presence of the silencing suppressor p19 and GFP expression was monitored under UV light from 3 to 12 dpi. All the vectors produced comparable levels of GFP expression in inoculated leaves at 5 dpi (data not shown). At 7 dpi, systemic GFP expression was observed for all the vectors; however, there were larger areas of fluorescent tissues in apical leaves of plants inoculated with PepGFPm2 than with PepGFPm1 or PepGFP2a (Fig. 2a, upper row). This difference increased after 12 dpi, when plants inoculated with PepGFPm2 produced the greatest amount of GFP fluorescence compared to PepGFPm1 or PepGFP2a (Fig. 2a, lower row). An RT-PCR analysis was carried out using primers to amplify a 1190 bp cDNA fragment for vectors carrying the *GFP* gene and a 476 bp one for the wild type virus. We sampled inoculated and systemic leaves at 7 dpi, and systemic leaves at 12 dpi. Results revealed the presence of *GFP* fusions at 7 and 12 dpi in all samples. Faint wild type *CP* bands were observed for PepGFP2a at all times post inoculation, and for PepGFPm1 and PepGFPm2 only at 12 dpi (Fig. 2b). The genetic stability of the most promising candidate, PepGFPm2, was tested over infection passages. GFP expression was detected during at least three passages (Fig. 3a) and an RT-PCR analysis of systemic leaves sampled at 10 dpi revealed the presence of the *GFP* fusion during the three passages (Fig. 3b).

In order to further improve our vector, the insert from pBIN61 was transferred to pJL89, a small and versatile binary plasmid [25], thus obtaining pJL89PepGFPm2. Infection with JLPepGFPm2 resulted in intense GFP fluorescence in agroinfiltrated leaves and evident GFP fluorescence in systemic leaves at 5 dpi, whereas PepGFPm2 produced only abundant although discontinuous GFP fluorescent spots in agroinfiltrated leaves and no fluorescence in systemic leaves by the same post-inoculation time (Fig. 3c). This suggested that pJL89PepGFPm2 was more efficient than pBPepGFPm2 for agroinoculations, favouring faster accumulation and spread of the tagged virus. In terms of stability through passages, both vectors behaved similarly (Fig. 3b). Next, PepGFPm2 and JLPepGFPm2 were tested in tomato plants. GFP fluorescent spots could be observed only sporadically in agroinoculated leaves and never in systemic leaves (data not shown). An RT-PCR assay to test insert stability showed that at 7 dpi, both wild type and fusion inserts could be detected in agroinfiltrated leaves, but at 12 dpi the wild type CP transcript was only detected (see below) indicating that the insert was lost. Thus, these new vectors improved GFP expression levels and stability in *N. benthamiana* but not in tomato plants.

### Expression of DsRed and mCherry from modified PepGFPm2 vectors

**Fig. 4:**
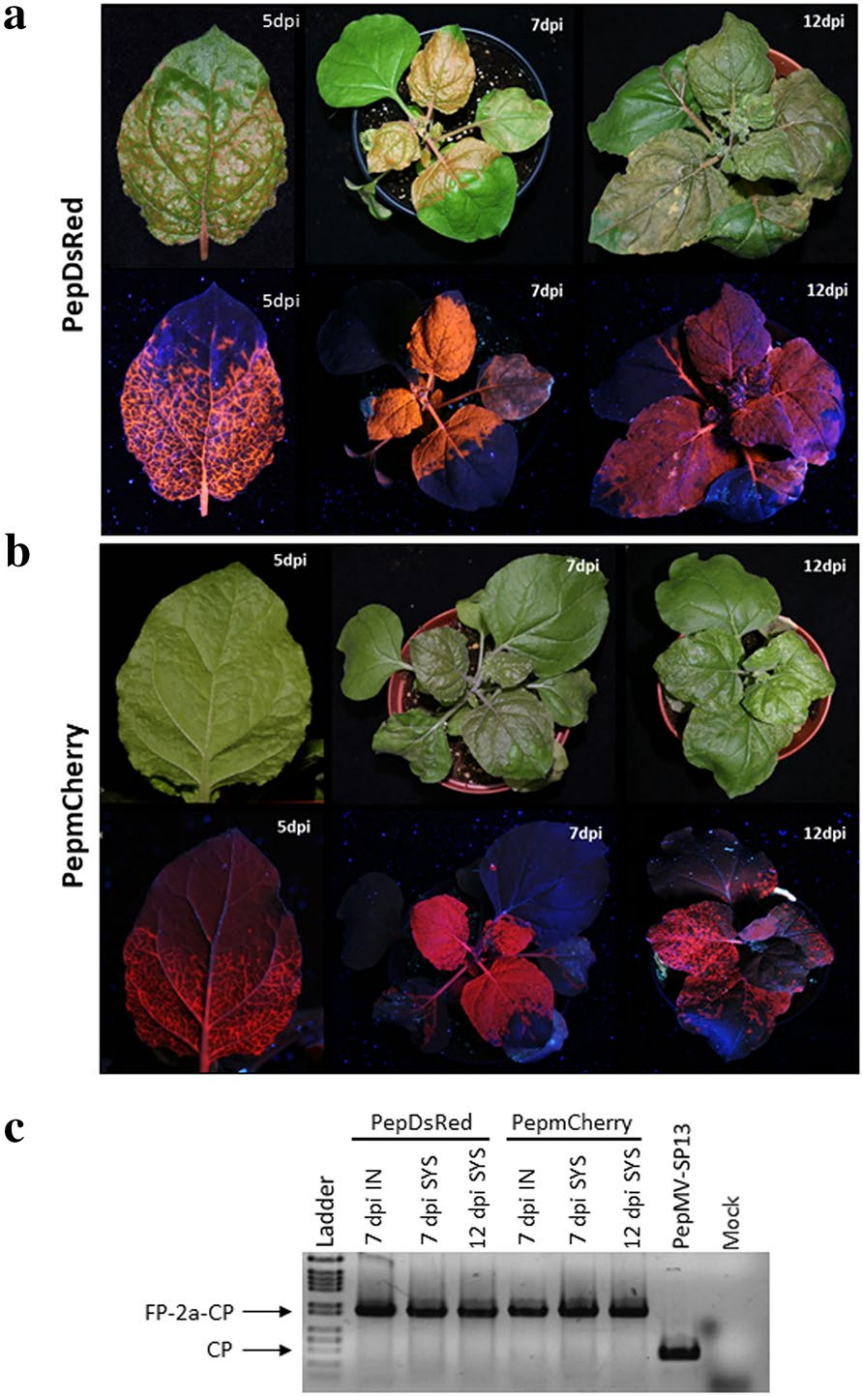
*N. benthamiana* plants infected with PepMV vectors expressing DsRed and mCherry. **a** Fluorescence in *N. benthamiana* plants inoculated with PepDsRed or **b** PepmCherry visualized under blue light and photographed with an orange filter at 5, 7 and 12 d post inoculation (dpi). **c** Agarose gel electrophoresis of RT-PCR products from individual plants to check insert stability in vectors infecting *N. benthamiana* plants at 7 and 12 dpi. A minimum of 6 plants were used per treatment, with each treatment replicated a minimum of 8 times. All infected plants showed systemic fluorescence by 7 dpi. IN: Inoculated leaves; SYS: Systemically infected leaves; FP: Fluorescent protein; PepMV-Sp13, wild type virus

**Fig. 5:**
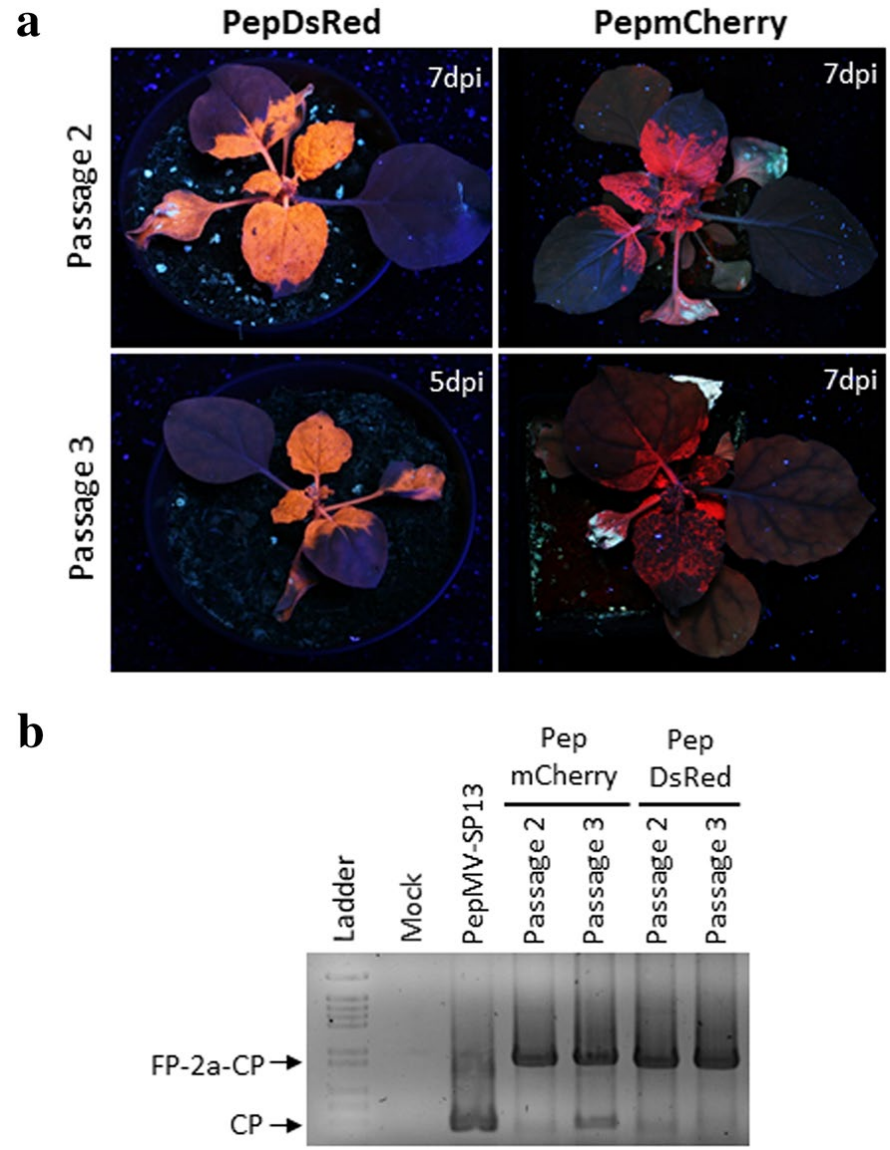
PepDsRed and PepmCherry stability in plant-to-plant passages. **a** DsRed and mCherry expression from PepDsRed and PepmCherry after the second and third passages at the times post inoculation (pi) indicated. **b** Agarose gel electrophoresis of RT-PCR products from individual plants to check insert stability at 7 dpi after passages. A minimum of 6 plants were used per treatment, with each treatment replicated a minimum of 8 times. All infected plants showed systemic fluorescence at 7 dpi. FP: Fluorescent protein; PepMV-Sp13, wild type virus

We hypothesized that a change of the reporter gene could confer more stability to the transgene in tomato plants. Thus, the *GFP* gene was replaced with the *DsRed* or *mCherry* genes in the pJL89PepGFPm2 backbone (Fig. 1b). After that, *N. benthamiana* plants were inoculated with PepDsRed and PepmCherry, and the plants were monitored over 2 weeks for fluorescent protein expression. At 5 dpi, it was already possible to observe an intense fluorescence along the veins in systemically infected leaves, and DsRed or mCherry expression was clearly visible to the naked eye. At 7 dpi, intense fluorescence could be observed in systemic leaves for the two vectors (Fig. 4a, b). At 12 dpi, reporter brightness started to decline for PepmCherry while fluorescence was as bright as before for PepGFPm2 and PepDsRed (Fig. 2a, 4a, b). An RT-PCR analysis of infected plant material was performed at different time-points. PepDsRed and PepmCherry should generate 1151 bp and 1184 bp products, respectively, while wild type PepMV should yield a 476 bp product. The presence of DsRed-2a-CP and mCherry-2a-CP sequence fusions was detected over time for both constructs and no wild type band was detected (Fig. 4c). An analysis of insert stability during the passages showed that DsRed and mCherry fluorescence was preserved for at least three passages, although mCherry expression became weaker in the third passage (Fig. 5a). Accordingly, an RT-PCR analysis of tissue sampled during passages at 7 dpi showed the product of the DsRed-2a-CP sequence fusion in all passages, but a wild type CP product appeared in the third passage for PepmCherry, indicating that this vector did eventually lose its integrity (Fig. 5b).

**Fig. 6:**
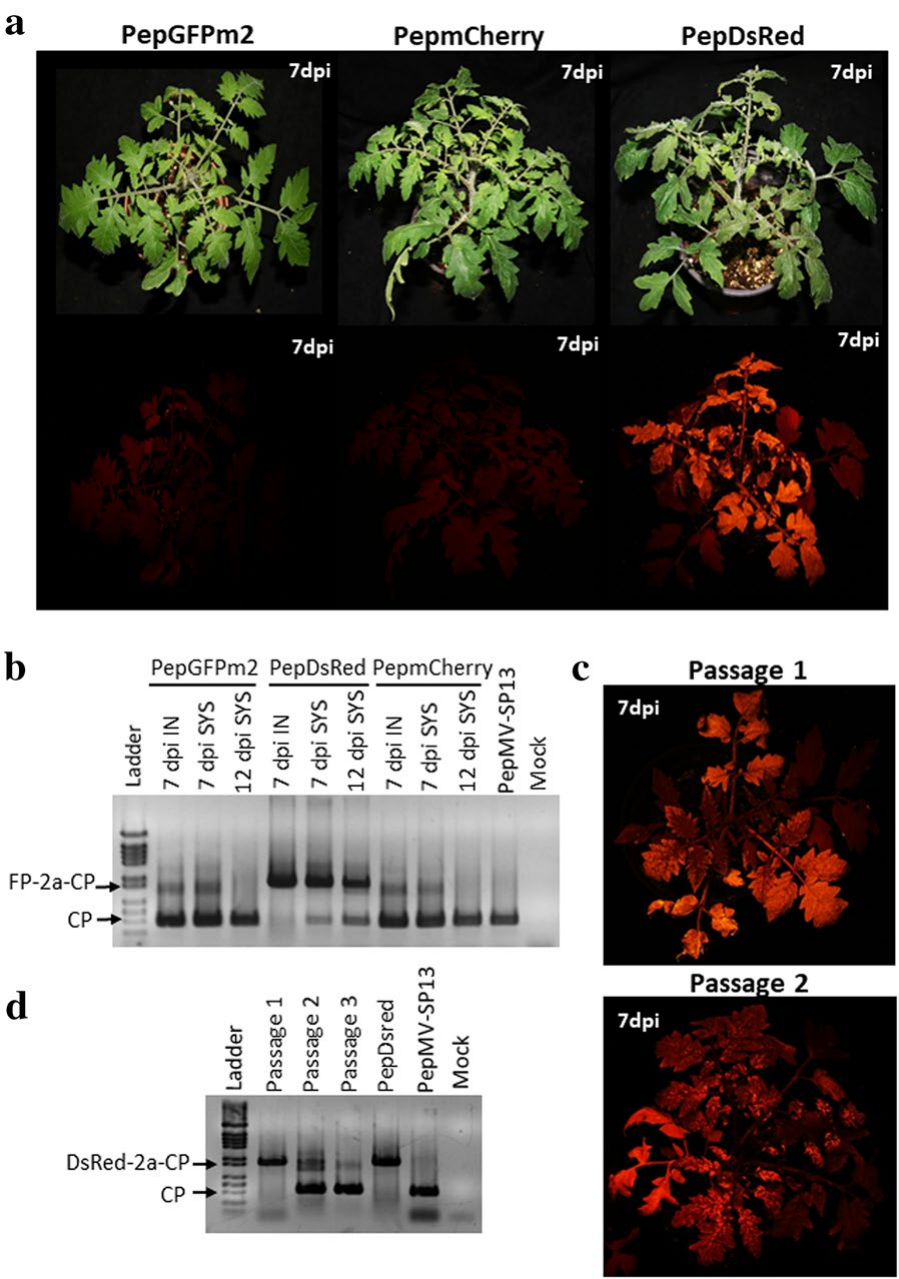
PepMV vectors expressing fluorescent proteins in tomato plants. **a** Fluorescence in tomato plants inoculated with PepGFPm2, PepmCherry or PepDsRed at 7 d post inoculation (dpi). **b** Agarose gel electrophoresis of RT-PCR products from individual plants to check insert stability in tomato at 7 and 12 dpi. **c** DsRed expression from PepDsRed after the second and third passages at 7 dpi. **d** Agarose gel electrophoresis of RT-PCR products from individual plants to check insert stability of PepDsRed in tomato passages at 7 dpi. A minimum of 6 plants were used per treatment, with each treatment replicated a minimum of 6 times. Only plants infected with PepDsRed showed systemic fluorescence; all of them in passage 1, 84% and none in passages 2 and 3, respectively.IN: Inoculated leaves; SYS: Systemically infected leaves; FP: fluorescent protein

After that, tomato plants were inoculated with PepGFPm2, PepmCherry and PepDsRed. We were able to detect DsRed fluorescence in agroinfiltrated and systemically infected leaves but neither GFP nor mCherry expression (Fig. 6a). An RT-PCR analysis confirmed that only the DSRed-2a-CP sequence fusion was stable over time in tomato, while GFP-2a-CP and mCherry-2a-CP sequence fusions were detected weakly at 7 dpi, with the signal for the CP wild type product being much more intense. At 12 dpi PepGFPm2 and PepmCherry had completely lost the insert while the DSRed-2a-CP product was still detectable (Fig. 6b). Passages were performed for PepDsRed, with fluorescence detected for up to two passages (Fig. 6c). An RT-PCR analysis showed that the vector was fully stable for the first passage, although from the second passage it was possible to detect the presence of the wild type CP product to a great extent (Fig. 6d).

In conclusion, the PepDsRed vector yielded robust and stable fluorescence protein expression in both *N. benthamiana* and tomato plants.

### Using the tagged vectors to analyse PepMV subcellular location

**Fig. 7:**
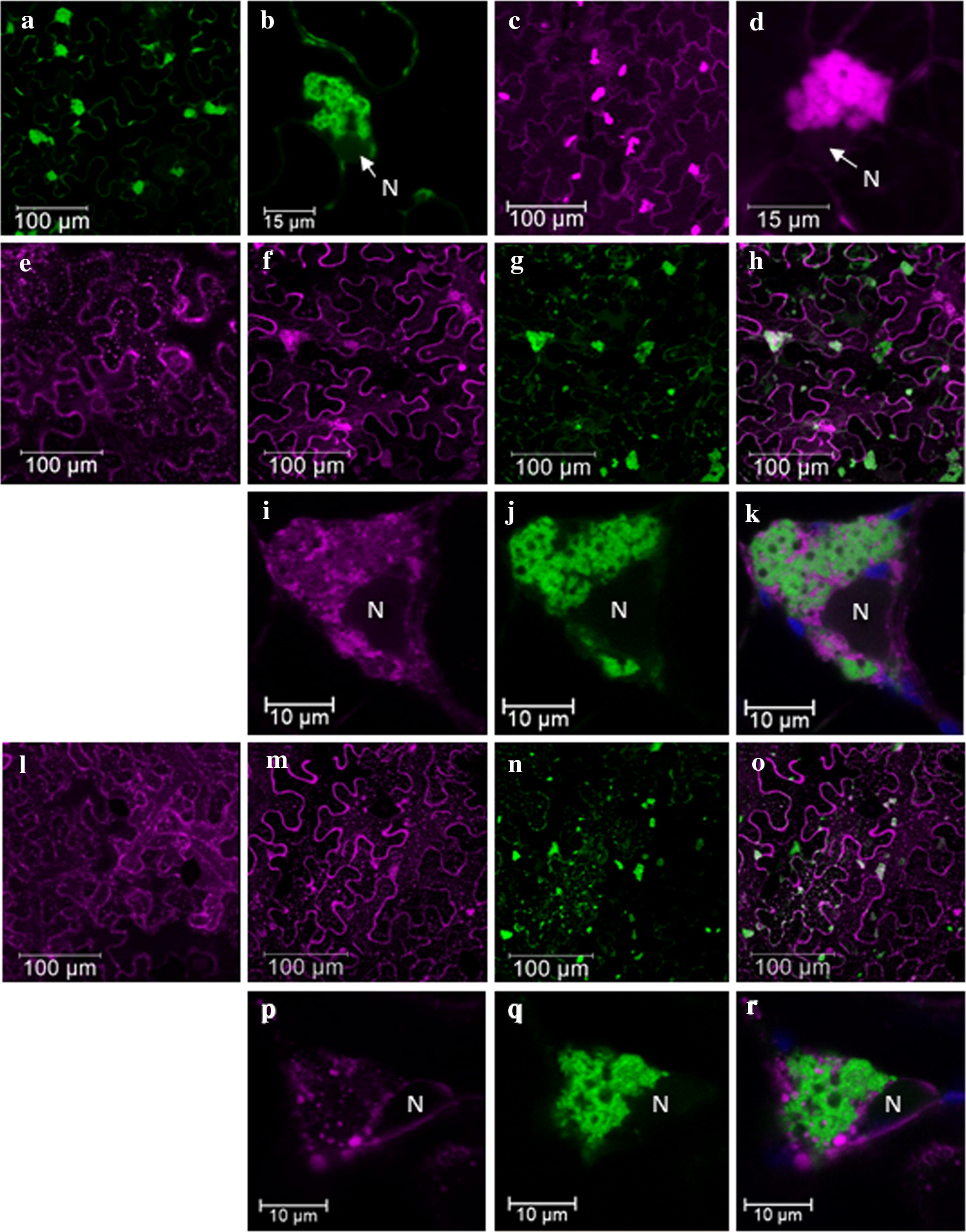
PepMV-induced subcellular bodies in *N. benthamiana* and tomato plants. **a** and **b** CLSM images of JLPepGFPm2 infection in *N. benthamiana* at 3 d post inoculation (dpi). **b** High magnification of a fluorescent body in JLPepGFPm2 infection to show its cytoplasmic localization and spatial relation with the nucleus. **c** and **d** CLSM images of PepDsRed infection in tomato at 3 dpi. **d** High magnification of a fluorescent body in PepDsRed infection. **e** Distribution of the endoplasmic reticulum marker (ER-mCherry) at 3 dpi in *N. benthamiana* in the absence of PepMV infection. In the presence of JLPepGFPm2 infection: **f** changes of ER-mCherry localization were observed, **g** green fluorescent bodies of JLPepGFPm2 and **h** merged image of **f** and **g** to see the matching. High magnification images: **i** the distribution of ER-mCherry during the infection, **j** the PepMV subcellular body and **k** merged image of **i** and **j** to see the incomplete matching between the red and the green labelling in the body. **l** Distribution of Golgi-mCherry marker at 3 dpi in *N. benthamiana* in the absence of PepMV infection. In the presence of JLPepGFPm2 infection: **m** changes of Golgi-mCherry localization were observed (**n**), green fluorescent bodies of JLPepGFPm2 and **o** merged image of (**m**) and (**n**) to see the matching. High magnification images of: **p** the distribution of Golgi-mCherry during the infection, **q** the PepMV subcellular body and **r** merged image of (**p**) and (**q**) to see the incomplete matching between the red and the green labelling in the body. N, nucleus. Blue colour corresponds to chloroplasts autofluorescence

JLPepGFPm2 and PepDsRed vectors were used to study virus localization in *N. benthamiana* and tomato cells. *N. benthamiana* leaves were agroinfiltrated with each of the two vectors with the same results, while tomato plants were agroinfiltrated only with PepDsRed. Fluorescence emission was analysed under CLSM in agroinfiltrated (Fig. 7) and systemic leaves (data not shown) at 3 and 10 dpi, respectively. We observed GFP/DsRed fluorescent bodies in almost every cell in both *N. benthamiana* and tomato samples (Fig. 7a, c). In tomato we counted an average of 85 and 133 fluorescent bodies at 3 and 10 dpi, respectively, in an area measuring 755 μm^2^, and always one per cell. In *N. benthamiana,* there was an average of 62 fluorescent bodies at 3 dpi in the same area, and at 10 dpi we were not able to count them because the tissue was too damaged. These bodies seemed to have a cytoplasmic localization and to be in close proximity to the cell nucleus (Fig. 7b, d). To study their possible relation with cytoplasmic organelles such as the endoplasmic reticulum (ER) and/or the Golgi (G) apparatus, new experiments were conducted in *N. benthamiana* by using ER and G markers [26]. In the absence of PepMV infection, the ER-mCherry marker gave rise to a typical network-like punctuated signal distributed along the cytoplasm (Fig. 7e) [26]. In plants co-agroinfiltrated with ER-mCherry and JLPepGFPm2, an apparent reorganization of the ER was observed (Fig. 7f),with the ER-mCherry’s fluorescence mainly localized to aggregates that looked quite similar to the bodies described above for JLPepGFPm2 or PepDsRed infected cells (Fig. 7f–h). Indeed, the ER-mCherry fluorescence labelled the same virus-induced bodies as the GFP fluorescence (Fig. 7i, j). However, the signals did not perfectly match (Fig. 7k). In a similar analysis using a Golgi-mCherry marker (G-mCherry) [26], an apparent change in the prevalent localization of the G-mCherry signal depending on the presence/absence of the JLPepGFPm2 infection (Fig. 7l–o) was also observed. Thus, the G-mCherry expressed alone showed its typical punctuated presence spread along the cytoplasm (Fig. 7l) [26] but in cells co-agroinfiltrated with JLPepGFPm2, a significant proportion of the G-mCherry signal localized to the green fluorescent bodies described above (Fig. 7m–o). The mCherry fluorescence of the G marker (Fig. 7p) appeared around the aggregates labelled in green (Fig. 7q) but the signals did not match when observed with sufficient magnification (Fig. 7r). Thus, both ER and Golgi organelles seemed to be intimately related but not to be main constituents of the bodies formed as the consequence of PepMV infection.

### A selectable PepBar vector for high-throughput screenings of tomato

**Fig. 8:**
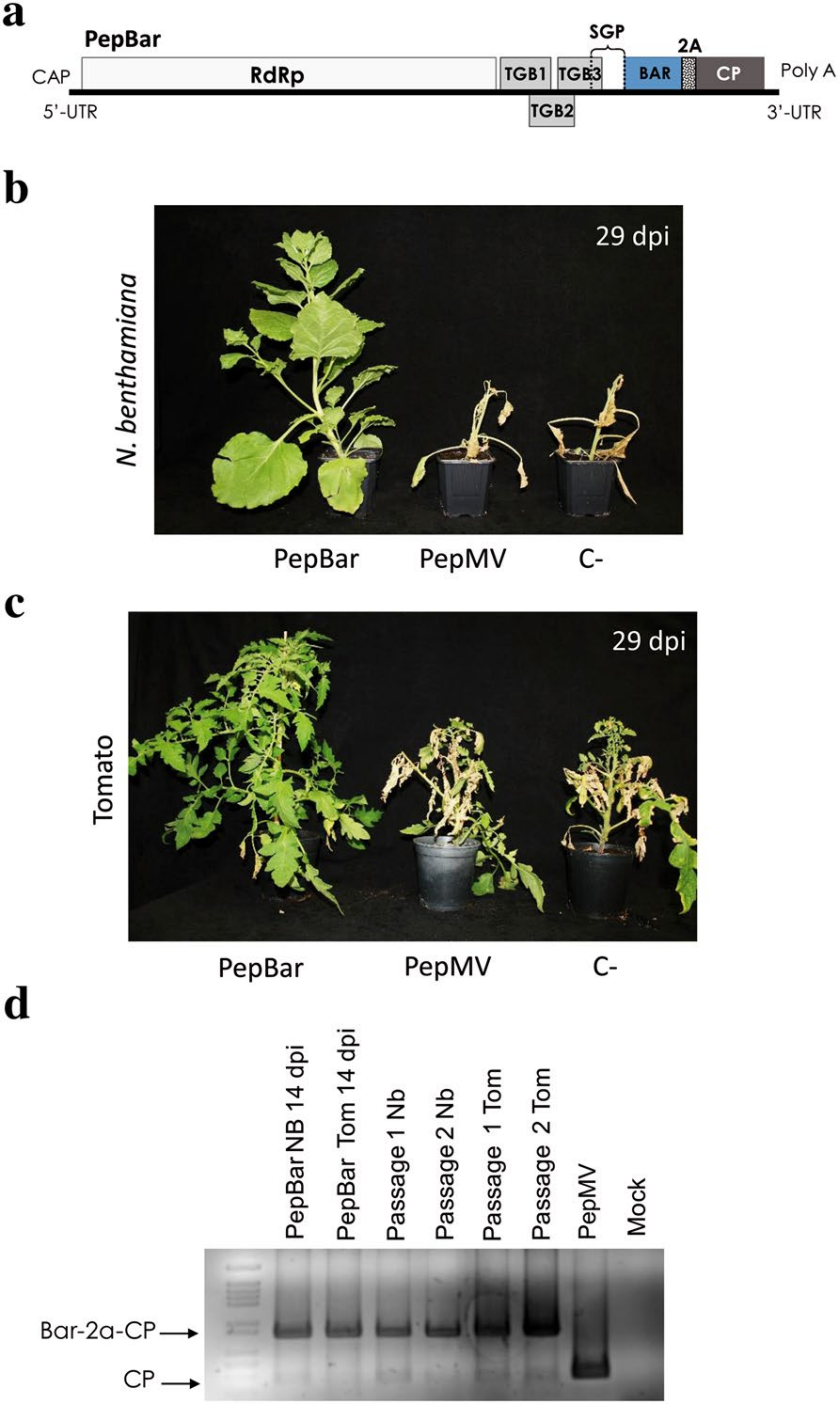
*N. benthamiana* and tomato plants infected with the PepMV vector carrying the *BAR* gene. **a** Schematic representation of the PepBar genome. **b** and **c** *N. benthamiana* and tomato plants inoculated with PepBar or PepMV-Sp13 (wild type virus); photo taken 2 weeks after herbicide treatment. **d** Agarose gel electrophoresis of RT-PCR products from individual plants to check insert stability in *N. benthamiana* and tomato plants at 14 d post inoculation (dpi). A minimum of 12 plants were used per treatment, with each treatment replicated a minimum of 3 times. Phenotypes described in the pictures were fully representative of results for the replicates. RdRp: RNA dependent RNA polymerase; TGB1, TGB2 and TGB3: Triple gene block; CP: Coat protein; SGP: CP subgenomic promoter; PepMV wild type virus

While PepDsRed seemed to be a sufficiently efficient vector for tomato, a new vector expressing the herbicide resistance *BAR* gene was considered for completing the set of PepMV-based tools, particularly as a selection tool for massive susceptibility screenings. pBPepBar was constructed with apBPepGFPm2-like backbone (Fig. 1a), with *GFP* substituted with *BAR*, the ATG of the *CP* mutated to AGG and the consensus Kozak sequence added just upstream from the *BAR* coding sequence (Fig. 8a). This vector was used to inoculate *N. benthamiana* plants, while control plants were inoculated with wild-type virus. At 15 dpi, all the plants were treated with a solution containing 0.05% glufosinate-ammonium (GA). One week after herbicide treatment, plants that were inoculated with the wild-typed virus showed severe wilting symptoms while plants inoculated with PepBar remained healthy (data not shown). After 2 weeks, all the control plants were dead while plants inoculated with PepBar remained healthy and vigorous (Fig. 8b). The presence of the BAR-2A-CP sequence fusion was monitored at several time points with RT-PCR. This analysis revealed that at 14 dpi the vector was stable, as found during two successive passages. *N. benthamiana* systemically-infected leaves were used to mechanically inoculate tomato plants, which were treated with 0.05% GA after 15 dpi. Two weeks after the treatment, the controls had completely wilted while the PepBar-infected plants were still alive (Fig. 8c). An RT-PCR analysis indicated that in tomato plants the BAR-2A-CP fusion was stable at 14 dpi. Vector stability was analysed by passaging the virus in tomato, showing that the vector was stable for at least two passages (Fig. 8d).

In summary, the new PepBar vector seemed to be stable and efficient for selection in both, *N. benthamiana* and tomato plants, thereby becoming a new tool in the PepMV-based toolbox.

## Discussion

A series of PepMV-based vectors had previously been constructed and tested, and this included PepGFP2a, a GFP-expressing vector that was very stable in *N. benthamiana* but unstable in tomato plants [11]. PepGFP2a expresses GFP and CP as a N-terminal translational fusion linked by the autocatalytic 2A peptide, and had a duplication of 36 nt encoding the N′-terminal region of the CP [11]. This strategy had previously been used successfully for the creation of a viral vector based on PVX, where the expression of the fusion peptide resulted in the accumulation of significant amounts of the CP fused to the fluorescent protein and lower amounts of the wild type CP necessary for correct encapsidation [27]. Later on, this strategy was used for the creation of vectors based on cowpea mosaic virus (CPMV) [28], bean pod mottle virus (BPMV) [29], wheat streak mosaic virus (WSMV) [30], plantago asiatica mosaic virus (PlAMV) [31] and tobacco mosaic virus (TMV) [32]. An alternative strategy would have been to fuse 2A-GFP to the C-terminus of the CP, but we tried that for our first versions of the PepMV vectors [11] and fluorescence did not spread efficiently in inoculated plants (data not shown). To solve the instability problem of PepGFP2a in tomato, we first worked under the hypothesis that instability could be caused by homologous recombination between repeated sequences during replication, as described in other similar cases [33–37]. Therefore, two new vectors were prepared, PepGFPm1 and PepGFPm2; the length of the duplicated fragment was reduced to 18 nt in PepGFPm1, with no consecutive nucleotide duplication in PepGFPm2. Of the two new vectors, PepGFPm2 was the most stable during passages in *N. benthamiana* plants, in agreement with results described by Draghici and Varrelmann [38] using PVX, where the frequency of transgene loss was conditioned by the length of the homologous sequence. Also, Nagy and Bujarski [39] described that the duplication of just 15 nt could activate homologous recombination in brome mosaic virus (BMV). However, even after the results obtained in *N. benthamiana*, the expression of GFP in tomato was observed only sporadically, perhaps due to the strong negative selection against *GFP* in tomato, as described for tomato bushy stunt virus (TBSV) [40, 41]. Work done also with TBSV concluded that the expression level of a foreign gene from a viral vector depended on the host into which the vector was inoculated [42]. Thus, two new vectors were designed expressing DsRed or mCherry, which were similar in size to GFP. The two new vectors were stable during successive passes in *N. benthamiana* plants, but only PepDsRed was stable in tomato. With PepDsRed an interesting new tool was built, which was useful for both macroscopic and microscopic observations at the cellular or subcellular level in tomato. Next, *N. benthamiana* leaves were inoculated with PepGFPm2 and tomato leaves with PepDsRed to study the subcellular location of PepMV in both hosts. Inoculated tissues were observed using CLSM at 3 and 10 dpi. Already at 3 dpi, most epidermal cells in both hosts showed PepMV-specific bodies marked with fluorescent proteins, and were found generally adjacent to the nucleus and one per cell. Note that an important proportion of the fluorescent proteins should be free [11], but local aggregation of the fluorescence was significant, implying either that free fluorescent proteins remain within these structures, or that aggregation of the CP fusion protein is such that PepMV-specific bodies are brighter than any other structure within the cell. Structures with similar features to these had previously been observed in infections with other viruses and referred to as viral replication complexes (VRCs), often described as viral factories [43–46]. VRCs could be considered as quasi-organelles responsible for coordinating different processes during viral infection such as replication, protein expression, virion assembly as well as intercellular transport [16]. For PVX, there is extensive knowledge about the VRCs generated during infection; the PVX “X-bodies” [17, 47] are built by some of the viral proteins in conjunction with cellular structures or organelles such as the ER or Golgi apparatus [16]. To analyse if there was a relationship between these organelles and the fluorescent bodies detected in cells infected with PepMV, an assay was carried out in which PepGFPm2 was co-agroinfiltrated with ER and Golgi apparatus markers, and the distribution of fluorescence was observed by CLSM in agroinfiltrated epidermal cells. Already at 3 dpi, it was observed that the ER and Golgi apparatus had been reorganized in the infected cells around the PepMV-induced bodies. This suggests a mechanism of recruitment of these membranous organelles to VRCs, perhaps as a part of a mechanism of protection of the virus factory against the defence mechanisms of the plant, by creating a safe space in which the viral RNA would not be recognized and degraded by the RNA silencing machinery of the plant, and/or as a mechanism to increase the VRC surface, possibly making replication more efficient [46, 48–51]. Further experiments are clearly required, but these observations strongly suggest that the fluorescent bodies detected in both *N. benthamiana* and tomato cells are the PepMV VRCs. Aggregates formed by PepMV particles perhaps related to our observations have been described in infected cells using transmission electron microscopy (TEM) [24].

To complete the toolbox based on PepMV, a vector was built for the expression of the *BAR* gene for glyphosate resistance; insertion of *BAR* in the PepMV genome proved to be stable in tomato and provided efficient expression of the transgene and a means of selection. In fact, this vector has been used successfully for carrying out susceptibility tests to PepMV in tomato (data not shown).

## Conclusions

We have significantly expanded the repertoire and improved the stability of viral vectors based on PepMV. This was particularly important for tomato, a crucial host for which not too many efficient vectors were available. We are confident that these vectors will be extremely useful for biotechnological applications [52], but also for the advancement of knowledge on the mechanisms and processes underlying virus/hosts interactions. For instance, by using these vectors, we have described, for the first time, the subcellular bodies that result from PepMV infection, which are likely the PepMV VRCs, opening new venues of research on the potexvirus/hosts molecular interactions.

## Methods

### Construction of PepMV-based vectors and mutants

We used overlapping PCRs [53] for the construction of PepGFPm1 and PepGFPm2. PepGFPm1 and PepGFPm2 fragments were amplified using pBPepGFP2a [11] and PepGFPm1 as templates, respectively. The fragments with the mutations were synthesized using primers CE-Pep303 (5′-CGGAATTGCAGGCACTGGG-3′) as forward primer for both constructs and CE-715RC (5′-**T**GG**C**GG**A**GC**G**CT**G**GA**T**GTGGCAGCAACAGGTGTTGT-3′) and CE-717RC (5′-**A**GC**G**GC**T**AC**T**GG**A**GT**G**GTGTCAGGCCCAGGGTTGGA-3′) (bold type represents silent mutations and underlined nucleotides the overlapping regions) as reverse primers for PepGFPm1 and PepGFPm2, respectively. The 3′-fragment, covering the *CP* gene and the 3′-UTR, was amplified with CE-43 (5′-GGGGTACCGCGGGCCCGGG(T)20-3′) as reverse primer for both fragments and the reverse complementary form of CE-715RC and of CE-717RC for PepGFPm1 and PepGFPm2, respectively, as forward primers. The overlapping PCR fragments were cloned into the pTOPO (Thermo Fisher Scientific) vector to generate pTOPOm1 and pTOPOm2. After that, pTOPOm1 and pTOPOm2 were digested with *Xma*I and *Xho*I, the resulting fragments were gel-purified and ligated into pTXLPepXL6 giving rise to pTXLPepGFPm1 and pTXLPepGFPm2. Lastly, pTXLPepGFPm1 and pTXLPepGFPm2 were digested and the resulting fragments carrying PepGFPm1 and PepGFPm2 were subcloned into pBIN61 [54] or pJL89 [25], which are binary vectors for expression in plants. For pJL89PepDsRed and pJL89PepmCherry constructs, overlapping PCRs were also used. Three overlapping DNA fragments were amplified in separate PCRs. The first one, a DNA fragment covering the 5′-UTR, the replicase, the *TGB* genes and the first 36 nt of the *CP* gene, was amplified using pBPepGFPm2 as template. CE-1955 (5′-CATTTCATTTGGAGAGGGAAAACAAAATAAATAAATAAATATACAAA-3′) was used as the forward primer for both constructs, while CE-2049 (5′-GATGACGTTCTCGGAGGAGGCCCTACTTGAAGTGGCAGCAACAGGTG-3′) was used as the reverse primer for pJL89PepDsRed and CE-2089 (5′-ACTTGAAGTGGCAGCAACAGGTGTTGTGTC-3′) for pJL89PepmCherry (the underlined primer sequences correspond to the overlapping regions). The *DsRed* and *mCherry* genes were amplified in a second PCR using pEGB 35S:DsRed:Tnos (Addgen plasmid # 68220) [55] and ER-rk CD3-959 [26] plasmids as templates, respectively. Primers CE-2048 (5′-ACTTCAAGT**AGG**GCCTCCTCCGAGAACGTCATC-3′) and CE-1861 (5′-CTTAAGAAGGTCAAAATTCAGGAACAGGTGGTGGCG-3′) were used to PCR-amplify the *DsRed* gene, while CE-2090 (5′-ACTTCAAGT**AGG**GTGAGCAAGGGCGAGG-3′) and CE-2091 (5′-TAAGAAGGTCAAAATTCTTGTACAGCTCGTCC-3′) were used to PCR-amplify the *mCherry* gene (the underlined primer sequences correspond to the overlapping regions). *DsRed* and *mCherry* start codons were removed by changing ATG to AGG (in bold type in primer sequences) to obtain a fusion protein in the same reading frame as the viral protein. The third PCR was performed to PCR-amplify the 2A sequence, the *CP* gene and the 3′-UTR using pBPepGFPm2 as the template and CE-1954 (5′-ATGCCATGCCGACCC(T)_50_-3′) as the reverse primer for both constructs, and CE-1860 and CE-2092 as the forward primers for pJL89PepDsRed and pJL89PepmCherry, respectively. Fragments resulting from the first and the second PCRs were mixed and amplified in a full-length DNA fragment using CE-1955 as the forward primer for both constructs and CE-1861 as the reverse primer for pJL89PepDsRed and CE-2091 for pJL89PepmCherry. The Gibson Assembly cloning kit (New England Biolabs) was used to join the full-length PCR fragments and the pJL89 vector according to the manufacturer´s instructions. For the pBPepBar construct, the *BAR* gene (coding for phosphinothricin acetyltransferase) was PCR-amplified from plasmid pFGC5941 (GenBank Accession No. AY310901). The PCR was done using primers CE-932 (5′-CGG*ACCGGT*GCCACCATGAGCCCAGAACGACGCCCG-3′), containing an *Age*I site (italics) and the consensus Kozak sequence (underlined), and CE-933 (5′-GGA*AGCGCT*TTTGATCTCGGTGACGGGCAG-3′) containing an *Afe*I site (italics) and the stop codon was removed. Note that the Kozak sequence was inserted just upstream of the *BAR* coding sequence and downstream of the minimal promoter of the CP subgenomic RNA [11].The resulting product was cloned in the pGEM-T easy (Promega) vector to generate pGEMBar. Then, pGEMBar was digested with *Age*I and *Afe*I and the resulting fragment carrying the *BAR* gene was gel-purified and inserted instead of the PDS fragment into a version of pTXLPepPDS2a [11] resulting in pTPepBar; the receptor vector had the *CP* ATG mutated to AGG and the duplicated CP subgenomic RNA sequence mutated as in PepGFPm2 (Fig. 1a). Lastly, pTPepBar was digested, and the resulting fragment carrying the PepBar construct was introduced into the *AgeI* site of the binary vector pBIN61 [54].

### Plants, inocula and fluorescence visualization

*Nicotiana benthamiana* and tomato cultivar (cv.) M82 plants were grown in a growth chamber set at 25 °C and 16 h/8 h (light/dark) conditions. Two weeks-old *N. benthamiana* plants were used for agroinoculations. For this, PepMV constructs were transformed individually into *Agrobacterium tumefaciens* strain C58C1. Overnight cultures were pelleted through centrifugation at 5000×*g* for 8 min and resuspended in an induction solution (10 mM MES, pH 5.5, 10 mM MgSO_4_ and 100 µM acetosyringone) for 3 h. PepMV cultures were mixed at a 1:3 ratio with *A. tumefaciens* cultures transformed with the pBp19 vector. Leaves of *N. benthamiana* plants were infiltrated with cultures (OD_600_ = 0.5) using needle-less syringes. Three weeks-old tomato plants were mechanically inoculated with homogenates of infected *N. benthamiana* systemic leaves in 30 mM sodium phosphate pH 8. Tomato leaves were rubbed with inocula and carborundum. GFP and mCherry fluorescences were monitored daily using a handheld UV lamp (Blak Ray B100-AP lamp, UV products, Upland, CA 91786, USA) while DsRed fluorescence was detected with blue light and an orange filter using a SafeCloner, a device originally designed to visualize EtBr, SYBR Green and SYBR Safe stained gels (Clever Scientific, www.cleaverscientific.com). Pictures were taken with a Canon EOS 400D camera.

### Confocal laser scanning microscopy

Small pieces of agroinfiltrated leaves were mounted onto glass microscope slides and CLSM imaging was performed with a Leica SP8 inverted confocal microscope. The scanning was done by using two objectives, a 40 × magnification oil immersion lens and a 63 × magnification glycerol immersion lens, and the excitation wavelengths used were 488 nm for GFP and 561 nm for DsRed and mCherry. Sequential imaging was used for samples co-expressing GFP and mCherry fusion proteins. Microscope power settings were adjusted to optimize contrast for each sample. For the CLSM images, we have represented the mCherry fluorescence in magenta.

### RNAs preparation and analysis

Total RNA was isolated using Tri-Reagent (Sigma Chemical Co., St. Louis, MO) and 1 µg of total RNA was used for RT-PCR analyses. The RT step was performed with Expand Reverse Transcriptase (Roche) according to the supplier’s instructions using CE-43 as the primer. After cDNA synthesis, the region between *TGB3* and the *CP* was amplified using primers CE-2308 (5′-CCATTGTCAGGCCATCATTGAC-3′) and CE-2309 (5′-GAACTCTGCACATCAGCATATGC-3′). DNA products were resolved by 1% agarose gel electrophoresis and stained with ethidium bromide.

### Herbicide treatment

Two weeks after the inoculation with PepBar, infected plants were treated with the herbicide Finale (BAYER CropScience, Victoria, Australia), which contains glufosinate-ammonium (GA) as active ingredient at a final concentration of 0.05% GA (w/v) in deionized water. Plants were photographed 15 d after the treatment.

## Data Availability

The raw data and materials generated and/or analysed during the current study are available from the corresponding author on reasonable request.

## Abbreviations

PepMV: pepino mosaic virus
CP: coat protein
dpi: days post-inoculation
RT-PCR: reverse transcription-polymerase chain reaction
CLSM: confocal laser scanning microscopy
ER: endoplasmic reticulum
G: golgi apparatus
GA: glufosinate-ammonium
VRCs: viral replication complexes
